# Legislation and Its Limitations in the Prevention of Crime and Disease

**Published:** 1901-11

**Authors:** John Bell Keeble

**Affiliations:** Nashville, Tenn.


ATLANTA

### Journal-Record of Medicine.

**Successor to Atlanta Medical and Surgical Journal, Established 1855, and Southern Medical Record, Established 1870.**

VOL. III. NOVEMBER, 1901. No. 8.

BERNARD WOLFF, M.D., EDITOR, Nos. 319–20 Prudential.

Published Monthly.

M. B. HUTCHINS, M.D., BUSINESS MANAGER, No. 64 Marietta St.

### *ORIGINAL COMMUNICATIONS.*

### LEGISLATION AND ITS LIMITATIONS IN THE PREVENTION OF CRIME AND DISEASE.*

BY HON. JOHN BELL KEEBLE,
NASHVILLE, TENN.

In the time allotted to this subject it is impossible to discuss at length the effect of legislation in the prevention of disease and crime. Therefore, in this paper only some limitations upon legislation in this regard will be touched upon. From the earliest records of civilized people, no successful crusade has ever been made against crime and disease except by the establishment of positive and prohibitive law. This law was the decree of a despot or the expression of a people through their chosen representatives, according to the nature of the government. In either event this was legislation, for legislation means nothing else than the establishment of law.

It may be said that the influence of law has never been imme-

*Read by invitation of Sociological Com. at meeting of Tri-State Med. Socy. of Ala., Ga. and Tenn., October 8, 1901, Nashville, Tenn.

diately successful, and its power grows from year to year and century to century, and the fact that perfect results are not at once obtained is no argument against the wisdom or necessity of enacting laws. In fact the study of all law impresses this upon every student, that obedience to law is a growth and evolution rather than a creation. And the reformer who seeks to suppress either crime or disease, must not expect a complete fruition of his plans in his lifetime. He is successful to an eminent degree, if he succeeds in embodying into a law that which the best wisdom of his contemporaries agrees should be enforced and which contains sufficient life to hold its place upon the statute books for generations to come. If he has done this, he may die with the assurance that the infringements against this law will decrease with the coming years, in proportion to the benefit that the people derive from a partial and imperfect, but increasing, enforcement.

That law can and does prevent crime and disease is too well established to need discussion and support.

The successful banishment of the scourges of cholera and yellow fever from the United States for so long a period—the confining of such plagues as smallpox to insignificant limits by compulsory vaccination and systems of quarantine—sets at rest the question that it does prevent disease as well as crime. The improved methods of sanitation in large cities, preventing fevers and diseases of various kinds that come from bad water, bad sewerage and filth in general, is a complete vindication of those who seek to make war against disease by legislation.

If there is one thing above another that makes the scientist sick at heart, it is to see the partial failure of his plans and hopes, brought about by the people whom he is striving to serve. And yet, when he bears in mind that no great benefactor of humanity ever lived who was not shackled by popular prejudice and the limitations of popular law, he should not be discouraged, for the real Savior of humanity—religiously, morally and physically—must learn that he will probably be crucified upon the cross of popular prejudice before the real merits of his reform are realized by the world.

But it will not do for a reformer to accept this as a fact, and, giving his creed to the world let it solve its own cause. It is now become his business to follow his delivery with a careful study of the limitations that surround it, with a view to spending some part of his remaining life in actively breaking them down, in order to hasten the day when the full benefits of his teachings may be given to the world.

Then it becomes necessary to consider some of the limitations that hinder and sometimes destroy the effects of benevolent legislation.

The main limitation upon legislation in the prevention of crime is the weakness and wickedness of humanity. No system of law can ever be devised that will completely prevent all those who are criminals at heart from offending against the dearest rights of the people, individually and as a State. It is true that legislation not only punishes the criminal and holds in awe many who are contemplating crime, but also it educates the community into a better moral status. By setting the seal of the government's disapproval, it quickens many a dull conscience and marks the way to morality and good citizenship for the weak-kneed traveler. Yet, evil is still born into the world, and the jail, the penitentiary and the halter are filling a mission, the end of which no man can prophesy.

The only remedy for this is the development of the moral natures of mankind, and, while legislation contributes its part in this educational work, there is by far the greater part that is to be accomplished otherwise.

Aside from this limitation, the limitations of legislation in the prevention of crime are identical with the limitations upon all legislation, the purpose of which is to prevent disease. To this branch of the subject the remainder of this paper is devoted.

The first limitation upon all such legislation is the weakness of the government. There is no question but in an absolute despotism law is more generally enforced than in any other form of government, and that as the government becomes more and more representative in form, it becomes more and more difficult to enforce law. As an instance of this in our own government, we see how hard it is to convict a man of prominence for crime committed against the State government, where judges, prosecuting attorneys, sheriffs and clerks are chosen by popular vote, and, on the other hand, how easy it is to convict such a man of crime in the United States Court, where all these officers are appointed, and where the judge holds office for life.

This being a representative government, we are met with this limitation upon legislation—a limitation from which we will never escape.

But the weakness of our government which concerns us more directly is the division of the United States into States, each, within certain limits, absolute sovereignties, and the existence not only of a dual government in every State—the national government and the state government—but also the great number of different States, so closely allied politically, socially and commercially; and each jealously guarding its constitutional rights. Therefore, each is proceeding with independent methods to prevent crime and to eradicate and crush out disease. It is needless to point out the difficulties that this condition presents. No quarantine system has ever been operated that will absolutely prevent the bringing of disease from one State into another.

Each State has ample power by p lice regulations to deal with diseases of all kinds, both by regulations that deal with local conditions and by quarantine laws.

One of the limitations, however, may be said to be the weakness of our national government in being unable to provide for the whole country a system of laws against the spread of disease. Tennessee might prohibit persons suffering from tuberculosis from marriage; Kentucky might not be willing to take such a stand. The diseased person marries. The husband and wife move to Tennessee, and the effects of Tennessee's laws are thus in a measure nullified.

The question presents itself, then, as to whether the best results will ever be reached in this regard until the States concede to the national government the right to legislate upon this subject for all the States. Whether this would be wise or not I do not pretend to say.

Another limitation upon successful legislation that our present system presents is that each city is granted the power, to a large extent, to make its own laws upon the subject of health. This, however, is an evil that each State by legislative enactment could correct.

A more dangerous limitation than all this is found in the fact that our legislative bodies are rarely composed of men who are abreast of the times from the standpoint of the science of health. It is hard to make the lawmaker understand why "A" should not be allowed to indulge in the privilege of a case of smallpox, if he desired to do so. But the real limitation of this kind upon legislation is the ignorance that makes the lawmakers enact impracticable laws—laws that do not appeal either to the reason of the people generally or to the wisdom of those who have mastered the situation. No law is effective, however needed, that fails to find response in the minds of reasonable people in general and the minds of special students of the question in particular. The history of all reformers is the history of give and take between the mass of the people and the reformers, and, no matter how great a scientist, no man is a great and useful reformer who fails to recognize this fact. Extreme legislation is legislation that the people will not stand, and no matter how desirable these measures may be, they are hindrances rather than helps to progress. With all respect I say that, as a general thing, physicians are the last men to recognize this fact. They have been so long accustomed to kill or cure in their own scientific style that, even in matters political, they find it hard to consider Patrick O'Donavan, of the 27th ward, in the enactment of law. And yet Pat must be reckoned with. The slow fight for the selection of men of knowledge and conservatism to places in legislative bodies is the remedy for this. Until this is done, much of disease will flourish, which might be prevented by wise and timely legislation.

The jury system is a most serious limitation upon legislation for the prevention of disease. I would not be misunderstood. Whatever may be the weakness of the jury system as put into practice, I am by no means unconscious of the magnificent part it has played in the preservation of the liberties and lives of the English speaking people. But, like all institutions, it has its drawbacks, and one of the most serious drawbacks is experienced in regard to the enforcement of laws, which embody the result of progressive thought and advanced wisdom—as the laws for the preservation of health. Under the constitutions in most, if not all, of the States, no man can be punished for any infraction of law, save by the consent of a jury, if the accused sees fit to demand one. This principle is even recognized in regard to offences against municipal law, and, although municipal courts are held by a Judge without the intervention of a jury, yet, by the right of appeal, the accused may secure a hearing before a jury and let them determine as to his guilt or innocence. If it is a difficult thing to get twelve men to agree upon a verdict in a case of larceny, which all agree is a crime, how much more difficult will it be to get a verdict of guilty in case of violation of a health ordinance. It is a difficult thing to get twelve men to agree that any man, for instance, should be fined for going into the bedroom of his child, sick with searlet fever, and then going at once to his place of work, perhaps carrying the germs of the disease to others. And yet, until this is done, there is a limitation upon all legislation looking to the prevention of disease and crime. It is almost a dream that we indulge in when we look to see the day in which a jury would punish either party to marriage, because, forsooth, the woman was already marked for the grave with consumption, or even had hanging over her some mental disease, that sooner or later would dethrone her reason, and yet, the survival of the fittest demands just this. Only when constitutions give to Boards of Health the power to determine all such questions, or leave such matters to juries composed of experts, will this limitation be removed. Perhaps a new day will break upon us to this extent: that is, that Judges will be more generally given power to instruct juries as to their verdicts. The jury system, as you know, is an English institution and stood between a people and a king. But, strange to say that this system became infinitely more powerful in this government than ever in England; and that England is today limiting the effect of the jury system by allowing Judges greater power, while we, without a King, are more loath to limit the system in any way. Until this matter is solved, there will always be a marked limitation upon all legislation for the prevention of disease and crime.

The last limitation upon legislation of this class is public sentiment. The influence of public sentiment will not, and should not, ever be lessened. No man can ever hope to make or enforce a law against which is a strong public sentiment. You cannot punish lynchers until the great body of the people demand such punishment. The eight-hour laws could never prove beneficial until public sentiment endorsed them. The enforcement of legislation against the employment of children of tender years in factories was the result of a new spirit of humanity that became public sentiment. The campaign against the old system of tenement houses is just begun, and landlords are becoming gradually amenable to law supported by public sentiment. Then, to break down this limitation means the development of a broader and better and more generous public sentiment. The first thing to be met and overcome is an exaggerated idea of personal liberty, an idea that in a country grown so populous in so short a time is yet hard to uproot.

The effort must be based upon two great principles: First, it must be based upon the great principle of selfishness. I and mine must be protected, and, in protecting me and mine, I, in common with all people, will surrender some of the prerogatives that I have been taught to believe to be inherent. Second, upon the great doctrine of "Thou shalt love thy neighbor as thyself," a principle generally regarded as quixotic, but which moves men most astonishingly when wisely aroused.

These are some of the limitations upon legislation in prevention of crime and disease. There doubtless are many more, but these may serve the purpose of this paper. Some of them are natural, pertaining to all people; some of them are artificial, pertaining to one form of government; but all of them, it seems to me, must be met and dealt with in the years to come.

You perform a great service when, as a physician and friend with an honorarium in view, you are called in to fight disease and death that are attacking one patient, but you perform a greater service when, impelled with a broad love of humanity, you come to take counsel as to best methods of meeting disease and death at the threshhold, rather than at the bedside. It is a greater service by so much as all broad affection for mankind is greater than affection for any one person.

